# Weight Loss Following Liraglutide Therapy in a Patient With Smith-Magenis Syndrome: A Case Report

**DOI:** 10.7759/cureus.111313

**Published:** 2026-06-22

**Authors:** Dave K Garg, Jeffrey Wei

**Affiliations:** 1 Internal Medicine, University of California, Los Angeles, Los Angeles, USA; 2 Endocrinology, Diabetes and Metabolism, University of California, Los Angeles, Los Angeles, USA

**Keywords:** genetic obesity, glucagon-like peptide-1 receptor agonist (glp1ra), hyperphagia, hypothalamic obesity, obesity, smith-magenis syndrome

## Abstract

This case reviews the use of a Glucagon-Like Peptide-1 Receptor Agonist (GLP1RA) in a young man presenting with excessive hunger and rapid weight gain compounded by an underlying rare form of genetic obesity, Smith-Magenis syndrome. The patient had, prior to presentation, seen an accelerated and persistent weight gain over the last five years despite attempts at portion control and behavior modification. Due to concerns of worsening metabolic disease, including prediabetes, dyslipidemia, and fatty liver disease, we started the patient on daily liraglutide and gradually titrated to the maximum dose over five months. He was able to tolerate the medication without severe gastrointestinal side effects, and alongside lifestyle and behavioral modifications, the patient lost 22.2 kg (49 lbs) over the course of 11 months. To our knowledge, there is only one other published case report documenting the use of GLP1RA therapy in a patient with Smith-Magenis syndrome.

## Introduction

Hyperphagia, defined as an abnormally increased appetite for and consumption of food, can occur due to hypothalamic injury, psychiatric disorders, certain medications, and genetic conditions. Patients diagnosed with forms of genetic obesity like Prader-Willi Syndrome (PWS) and, even more rare, like Smith-Magenis Syndrome (SMS), often struggle with hyperphagia from an early age [1]. In SMS specifically, haploinsufficiency of the RAI1 gene leads to decreased expression of proopiomelanocortin (POMC) and Brain-Derived Neurotrophic Factor (BDNF), thus impacting the leptin-melanocortin pathway in an unopposed manner to drive increased hunger. This dysregulated eating pattern leads to uncontrolled weight gain and serious cardiometabolic morbidities, which can be particularly challenging to navigate in these patients given the current limited modalities to correct or rebalance their drivers of satiety. With the advent of Glucagon-Like Peptide-1 Receptor Agonists (GLP1RAs), there has been a large interest in the utility of these medications for the management of obesity, with clinical trials showing an average weight loss of 15-20% [2]. However, there has been a near-total absence of GLP1RA data in SMS patients to date. Here we describe a case of a young man with SMS who was started on liraglutide (Saxenda®) as an adjunct to ongoing lifestyle and behavioral modification efforts.

## Case presentation

A 31-year-old male presented to the endocrinology clinic with his mother due to a persistent increase in his weight over the last several years. Given his intellectual disability, his mother, as his primary caretaker, served as historian on his behalf. He had been diagnosed with Smith-Magenis syndrome (SMS) at an early age by a specialist at Children’s Hospital Los Angeles, who confirmed a deletion of chromosome 17p11.2 in the setting of significant cognitive impairment, developmental delay, aggressive behavior, and recurrent ear infections. His mother noted that the patient was “always hungry” from a young age, often seeking large portions and wanting the “biggest size”. He would have a pattern of waking up at 2-3 am and eating at night when the rest of the family was sleeping. They had never seen a nutritionist prior, but his mother tried to control his portions and keep food away during the evenings, locking the fridge at night. Over the last several years, his primary care doctor became more concerned about the relatively quick weight gain, particularly given the early presence of metabolic disease.

At the time of presentation, his medical history was notable for borderline prediabetes with an elevated hemoglobin A1C (HbA1C) at 5.7% and dyslipidemia. Hepatic steatosis was appreciated on repeat abdominal ultrasounds. He presented at the highest weight of his life at 145.1 kg (320 lbs), with a body mass index (BMI) of 44.68 kg/m². Physical exam showed a young male with brachycephaly, short stature, small hands and feet, and apparent central adiposity. Endocrine evaluation did not reveal any abnormalities in his thyroid. There was low suspicion for Cushing’s disease; there was no evidence of Cushingoid facies, dorsocervical fat pad accumulation, or violaceous striae. The patient had never been sexually active prior.

**Figure 1:**
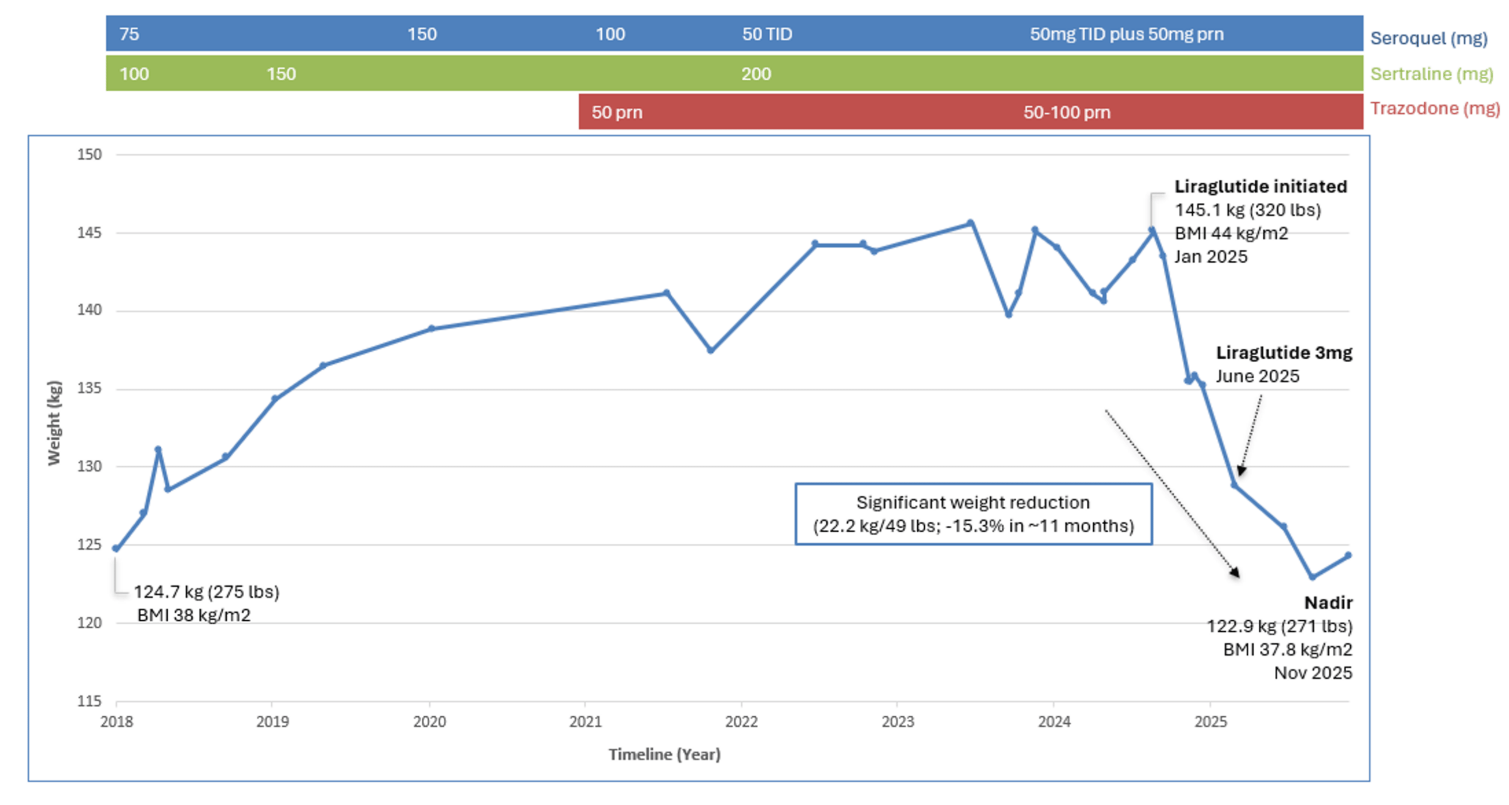
Graph of patient’s weight trend Figure 1 displays the patient’s weight trend from 2018 to 2026. Each data point represents a weight measured at a clinician’s office. The dose adjustments of the patient’s mood stabilizers over time are shown at the top. Liraglutide was initiated in January 2025, as noted; the patient was titrated to the maximum dose of liraglutide 3 mg daily by June 2025. BMI, Body Mass Index; TID, Three times a day; prn, as needed

He was actively following up with psychiatry for management of his mood disturbance, for which he was on several mood stabilizers, including seroquel, sertraline, and trazodone. The doses of these three medications were slowly titrated up through the years prior to and up to our initial consultation, as shown in Figure 1. No additional mood-stabilizing medications were introduced during these six years. Non-pharmacologic assistance techniques, including a reward system, limiting instructions to simple one-step instructions, and music therapy, were being discussed and implemented at home. Dietary recall revealed unstructured eating patterns, with days starting with large portions of processed foods such as cereal or multiple sweetened yogurts, skipped lunches, large portions of fruits, and portioned dinners by his mother, which often left the patient hungry. Exercise was limited to walking and hiking due to the patient’s inability to cooperate with more structured exercise. For several months, dietary modification was attempted with an emphasis on higher lean protein intake, dietary fiber from vegetables, balanced meals using a structured plate method, and minimal added sugars. Walking with weighted supports with the family was encouraged to increase his overall activity during the day. Despite these strategies, the patient’s weight decreased roughly 4.5 kg (10 lbs) before returning to his prior peak of 320 pounds in December 2024.

At this time, his mother expressed interest in pursuing GLP1RA therapy. Counseling regarding a unique theoretical risk of gastric rupture in the setting of unchanged hyperphagic behavior and reduced gastric motility from the medication was discussed at length. Given this and the potential for increased nausea with newer, more potent GLP1RAs like semaglutide (Wegovy), it was jointly decided to start with a GLP1RA with a shorter half-life first, like liraglutide (Saxenda®) as his first trial. He was initiated on the lowest dose of liraglutide in January 2025 and, over the next several months, was cautiously increased to the highest dose, 3 mg daily. During this time, the patient did not endorse serious gastrointestinal side effects such as nausea, abdominal pain, diarrhea, or constipation, though assessment of some adverse events was limited due to the patient’s underlying cognitive impairment. His vital signs remained stable, with his heart rate between 60 and 70 beats per min during subsequent clinic visits. The patient’s eating patterns were evaluated using an SMS food-related problems questionnaire (SMS-FRPQ) created by Elatrash et al., with his mother noting little difference in satiety impairment and desire for food [3]; however, she observed he was more redirectable than previously, and portions had reduced to approximately 75% following liraglutide therapy and with her continued behavioral interventions. His weight steadily declined, reaching a nadir of 271 pounds in November 2025, with a 22.2 kg (49 lb) loss over 11 months (15.3% of body weight). His HbA1C improved. Cholesterol panel remained above normal limits. Pertinent laboratory results are included in Table 1.

**Table 1:** Laboratory values prior to and following the start of liraglutide GFR, Glomerular Filtration Rate; TSH, Thyroid Stimulating Hormone; HDL, High-Density Lipoprotein; LDL, Low-Density Lipoprotein

		Prior to liraglutide		Following liraglutide	
Laboratory Test	Reference	4/2024	10/2024	3/2025	10/2025
Glucose	65 - 99 mg/dL	-	91	80	87
Hemoglobin A1C	<5.7 %	5.7%	5.5%	5.4%	-
Creatinine	0.60 - 1.30 mg/dL	-	0.96	1.03	1.09
Estimated GFR	mL/min/1.73m2	-	>89	>89	>89
TSH	0.3 - 4.7 mcIU/mL	1.6	-	-	2.6
Triglycerides	<150 mg/dL	223	-	262	220
Total Cholesterol	mg/dL	245	-	247	263
Cholesterol, HDL	>40 mg/dL	35	-	36	35
Cholesterol, LDL, Calc	<100 mg/dL	164	-	159	184

The patient’s weight trend is shown in Figure 1, along with an approximate timeline of dose titrations for seroquel, sertraline and trazodone.

## Discussion

Smith-Magenis syndrome (SMS) is a rare autosomal dominant disorder caused by a deletion within chromosome 17p11.2 or due to a pathologic variant of Retinoic Acid-Induced 1 (RAI1) [4,5]. As a complex multi-systemic syndrome, SMS causes extensive clinical sequelae including intellectual disability, congenital heart defects, genitourinary malformations, hearing loss, ocular abnormalities, and scoliosis [5]. While it shares features with those of other genetic obesity syndromes like Prader-Willi syndrome (PWS), a prominent feature of SMS is prognathism, with retrusion of the midface and protrusion of the mandible, which worsens with age. Behavioral disturbances are common and include aggression, tantrums, and self-injurious behavior [4]. Most patients are diagnosed around four to five years of age, wherein facial and behavioral features in the setting of dyslipidemia are often readily identified [6].

Elatrash et al. conducted a survey with caregivers of patients with SMS [7]. Nearly all reported that patients with SMS were obsessed with or perseverated on food. Patients lacked satiety cues, with over half of patients eating to the point of vomiting. Many caregivers resort to locking refrigerators or pantries; similarly, our patient’s mother would control his intake by locking the fridge at night. While medications like mood stabilizers are often prescribed to manage these challenging behaviors and provide caregiver relief, they can inadvertently compound weight challenges. For instance, our patient has been on Seroquel, sertraline, and trazodone, which are known to cause weight gain as a side effect. Though we are unable to prove causality in this single case study, as there are several factors that likely contributed to his weight changes, there appears to be a significant weight increase following higher doses of these weight-gain-associated medications, as shown in Figure 1.

Traditional emphasis on balanced hypocaloric diets and exercise in patients with monogenic obesity is often inadequate for clinically significant and sustained weight loss and maintenance. Pharmacotherapy for obesity is emerging as a viable treatment modality, though there is less data to support its efficacy in treating genetic obesity. To our knowledge, there are no case reports to date regarding the effects of phentermine, phentermine-topiramate, or naltrexone-bupropion on patients with SMS. Setmelanotide, a melanocortin-4 receptor (MC4R) agonist, is FDA-approved for several monogenic forms of obesity, including Bardet-Biedl syndrome and POMC, leptin receptor (LEPR), or proprotein convertase subtilisin/kexin type 1 (PCSK1) deficiencies. While studies demonstrated long-term, clinically significant, and sustained weight loss in these cohorts, unfortunately, setmelanotide was ineffective (only 0.28% weight loss) in a small open-label study of patients with SMS [8,9]. Bariatric surgery has yet to be shown as a long-lasting therapy for treating genetic obesity [10].

GLP1RAs have been shown to target key areas in the brain that modulate reward processing, impulse control, and emotional regulation [11]. In addition to demonstrated cardiometabolic effects, there is literature to suggest neuroprotective effects of GLP1RA that could be especially beneficial in patients with early neurocognitive disease [12]. These multifaceted effects suggest that GLP1RAs may offer unique, broad-spectrum utility in addressing the complex interplay between metabolic dysfunction and neurodevelopmental deficits seen in syndromic obesity. At the same time, a significant theoretical concern regarding GLP1RA therapy is an elevated risk of gastric rupture [13]. This vulnerability is possibly increased when GLP-1RA-induced gastric slowing is coupled with these patients’ impaired pain perception and tendency to eat past fullness [13]. It is for this reason that, when considering a GLP1RA trial in our patient, we opted for using an option with a shorter half-life in case there were any significant early complications when titrating the medication.

While emerging literature reviews the utility of GLP1RAs in genetic obesity syndromes, published clinical data remain scarce and demonstrate highly heterogeneous therapeutic responses. For instance, trials evaluating liraglutide and semaglutide in monogenic obesity reveal a broad range of weight-loss outcomes, spanning from a modest 4.7% to an exceptional 29.3% reduction, the latter observed in a Bardet-Biedl syndrome case report utilizing sequential liraglutide and semaglutide therapy [8,14,15]. Similarly, a single-case report of a patient with a heterozygous pathogenic MC4R variant demonstrated a robust 20% weight loss with tirzepatide [8]. Conversely, GLP1RA therapy in PWS has yielded mixed results, with some individuals achieving up to a 14.4% weight reduction while others exhibit no significant weight change [16,17]. Taken together, these highly variable outcomes underscore a limited evidence base and highlight the need for more studies to fully understand the broader efficacy of these new therapies in managing genetic obesity.

To date, there is only one other known study investigating the role of GLP1RA in patients with SMS [18]. In their study, they describe an 18-year-old female with SMS and type 2 diabetes (HbA1C 7.8%) who was started on subcutaneous weekly semaglutide. Over two years, the patient had a total weight loss of 3.6 kg. Curiously, when Correia et al. transitioned their patient to daily oral semaglutide, the patient experienced weight gain alongside deteriorating behavioral appetite regulation [18]. While our patient had sustained weight loss following liraglutide therapy, objective measures of satiety impairment and hunger were largely unchanged on the SMS-FRPQ [3]; more cases are needed to better understand the efficacy and potential utility of GLP1RAs in treating patients with SMS. A key difference between our patient and Correia’s patient was the concurrent diagnosis of type 2 diabetes. Several studies have indicated that patients without diabetes achieve greater weight loss on GLP1RA therapy compared to patients with diabetes [19]. Thus, the absence of comorbid diabetes in our patient potentially increased their metabolic responsiveness to our chosen therapy, though larger studies are ultimately needed to validate these findings.

Despite clinically significant weight loss, it is notable that our patient did not have a concurrent reduction in cholesterol levels. Patients with SMS are at increased risk of dyslipidemia due to a deletion in the gene sterol regulatory element-binding transcription factor 1 (SREBF1) on chromosome 17, which regulates the low-density lipoprotein (LDL) receptor and cholesterol homeostasis [6]. It is possible that we are seeing mobilization of adipose cholesterol stores, resulting in a transient increase in LDL cholesterol during his active weight loss, and plan to continue to monitor his levels closely and consider statin therapy if needed [20]. Further research is needed to understand the full impact of weight loss pharmacotherapy in patients with SMS on their overall cardiometabolic health.

## Conclusions

Our patient with SMS was observed to have clinically significant weight loss following liraglutide therapy alongside lifestyle and behavioral strategies. While there were no apparent alterations in food behavior during liraglutide treatment compared to baseline, as indicated by the SMS-FRPQ, portion sizes were reduced through a combination of the medication and continued caregiver guidance. He was able to tolerate the maximum dose of therapy without major adverse side effects. Our observations of this single case cannot be generalized to all patients with SMS. The scarcity of similarly published case reports limits our understanding of the benefits and risks of GLP1RA therapy in this population. Further studies are needed to examine the short and long-term impacts of GLP1RA-based medications in this challenging patient population.
